# Has the Integrated Medical Insurance System promoted return-to-hometown entrepreneurship among migrant workers? Evidence from China

**DOI:** 10.3389/fpubh.2024.1323359

**Published:** 2024-02-02

**Authors:** Pengcheng Huang, Zhengxiu Sun, Linfang Li, Jia Li

**Affiliations:** ^1^School of Finance, Anhui University of Finance and Economics, Bengbu, China; ^2^School of Economics and Management, Southeast University, Nanjing, China; ^3^Jinan No.1 High School in Shandong Province, Jinan, China; ^4^School of Clinical Medicine, Chengdu University of Traditional Chinese Medicine, Chengdu, China

**Keywords:** Integrated Medical Insurance System, return-to-hometown entrepreneurship, migrant workers, pullback effects, urban–rural mobility

## Abstract

An important way to reduce urban–rural disparity lies in encouraging migrant workers to return to their hometowns for entrepreneurship. This paper examines the effect of the Integrated Medical Insurance System on the return-to-hometown entrepreneurship among migrant workers. Using microdata from the China Household Finance Survey (CHFS) spanning from 2013 to 2019, we find that the Integrated Medical Insurance System (IMIS) significantly increases the likelihood of migrant workers returning to their hometowns for entrepreneurship by 0.44%. This result remains stable after a series of robustness checks. Heterogeneity results indicate that this “pullback effect” is more pronounced for those who are male and with lower educational levels, higher income, larger social networks, and lower risk preferences. Finally, the interaction between the Mass Entrepreneurship and Innovation policy (MEI) and IMIS can create a more significant combined effect in promoting the return of migrant workers to their hometowns for entrepreneurial activities.

## 1 Introduction

In recent decades, developing countries, including China, have witnessed a pronounced trend of rural-to-urban migration. Migrant workers hold a crucial position in advancing economic development and constitute a central segment of the mobile population (1). The movement of these labor forces from rural areas has given rise to the phenomenon commonly referred to as “rural hollowing,” which has led to a dearth of rural labor, an aging demographic, and an increase in poverty levels (2). This phenomenon has contributed to the deterioration of rural regions and the further exacerbation of the urban–rural divide. Therefore, facilitating the return of migrant workers from their jobs abroad to their hometowns for work and life seems to be a potential solution.

The impact of welfare programs on population mobility and employment has garnered substantial attention from economists and policymakers (3). In general, individuals are more inclined to migrate to regions offering generous welfare benefits, a phenomenon commonly referred to as the “welfare magnet effect” (4–7). Medical insurance, as a crucial facet of social welfare programs, plays a pivotal role in alleviating population mobility stemming from employment-related factors, often referred to as “job lock” (8–12). It is noteworthy that these findings primarily originate from empirical studies conducted in developed countries, with limited evidence available for developing countries. Among the scarce studies based on Chinese cases, Shi (13) discovered that a new rural health insurance program initiated by the Chinese government in 2003, known as the New Cooperative Rural Medical Scheme (NCRMS), had negative effects on rural-to-urban migration mobility in China. Similarly, Wang et al. (3) revealed that the NCRMS reduced the likelihood of rural residents working in other regions but did not observe a significant “pullback effect” for migrant workers returning to their hometowns or nearby regions from elsewhere. However, it is crucial to recognize that these outcomes are indicative of the previous NCRMS, which, while effectively curbing the outflow of rural migrant workers through its residency-based healthcare insurance system, also impedes the return of laborers engaged in external employment. This situation is possibly detrimental to the goal of narrowing urban–rural disparities.

In 2016, the State Council of China issued the “Opinions on Integrating the Basic Medical Insurance Systems for Urban and Rural Residents,” with the overarching aim of reducing healthcare coverage disparities between urban and rural areas. This was achieved by merging the NCRMS and the Urban Resident Basic Medical Insurance (URBMI) into the Urban and Rural Resident Basic Medical Insurance (URRBMI). Compared to the previous NCRMS, the URRBMI offers higher reimbursement rates, expanded reimbursement ceilings, and a more streamlined reimbursement procedure, thereby significantly narrowing the disparity in healthcare benefits between urban and rural areas (14). This development raises an intriguing scientific inquiry: Can this plausibly exogenous policy alteration stimulate the return of rural laborers who work outside their hometowns? To the best of our knowledge, there is currently no existing literature that offers a more rigorous causal inference on this particular issue.

Several local governments across China have progressively undertaken the integration of URBMI and NCMS, culminating in the creation of the Integrated Medical Insurance System (IMIS). This presents a unique opportunity to investigate this matter within a Difference-in-Differences (DID) framework. Thus, we leverage data from the China Household Finance Survey (CHFS) spanning from the years 2013 to 2019, employing a DID approach to assess the impact of IMIS on migrant workers’ return to their hometowns for entrepreneurial pursuits. Our DID results reveal that IMIS significantly increases the likelihood of migrant workers returning to their hometowns for entrepreneurship by 0.44%. This finding remains robust following a series of rigorous tests, including DIDM, endogeneity, alternative models, and placebo tests. Heterogeneity results suggest that the pullback effect after the implementation of IMIS is more pronounced for those who are male and with lower educational levels, higher income, larger social networks, and lower risk preferences. Considering that the entrepreneurship policy, Mass Entrepreneurship and Innovation (MEI), coexists within the timeframe of our study, and after excluding its potential contamination on the outcomes, our moderating effect model shows that the interaction between the MEI and IMIS yields a more substantial combined impact in facilitating the return of migrant workers to their hometowns for entrepreneurial ventures.

We make the following marginal contributions: Firstly, our findings enrich the existing body of research on the policy impacts of the IMIS. Prior studies have provided valuable insights into the impact of IMIS, including its role in reducing overwork among migrant workers (15), enhancing the well-being of rural older adults (16), improving healthcare access and quality while lowering medical expenses (17), alleviating medical impoverishment (18), promoting healthcare equity (19–21), enhancing health status (17, 22), fostering social integration (23), and boosting employment (24, 25), as well as increasing the utilization rate of healthcare services among the rural migrant population (19, 26–28). However, in contrast to prior research focused on the impact of healthcare insurance on employment and health welfare, our study extends its scope to the entrepreneurial decisions of migrant workers. This expansion provides a novel theoretical framework for comprehending the dynamics of rural labor force. Our findings complement this existing body of work by highlighting its role in facilitating the return of migrant workers to their hometowns to initiate entrepreneurial ventures and observing heterogeneous responses within different subgroups. Furthermore, it is worth noting that the integration of URBMI and NCRMS into IMIS represents an enhancement of the social security system, effectively addressing several shortcomings of the previous NCRMS. Our findings align with the broader literature on the “welfare magnet effect” and “pullback effect,” where regions offering enhanced welfare benefits tend to attract population inflows (29, 30).

Secondly, our study makes a significant contribution to the field of entrepreneurship determinants. Prior studies have extensively explored the multitude of factors that influence individuals’ decisions to embark on entrepreneurial ventures, including individual characteristics (31, 32), household attributes (33, 34), the prevailing economic landscape (35, 36), and the cultural milieu (37). However, it is worth highlighting that, to the best of our knowledge, there is a relative scarcity of studies that offer more rigorous causal inferences from the perspective of the health insurance system. Our study is an important supplement to this field.

Thirdly, our findings hold significant implications for policymaking and practice. They underscore the potential of improving healthcare insurance system in promoting the return of migrant workers to their hometowns for entrepreneurial endeavors. Additionally, our study reveals a valuable policy synergy between healthcare coverage and policies aimed at fostering rural entrepreneurship. To some extent, this could reflect the great potential of optimizing healthcare insurance in addressing pressing issues such as rural depopulation, the urban–rural divide, the enhancement of rural healthcare welfare, and the promotion of balanced economic development. Consequently, for countries grappling with similar challenges involving disparities in urban–rural healthcare welfare and the outflow of rural populations, China’s experience may offer a feasible solution.

The remainder of the paper is organized as follows: Section 2 introduces the IMIS and migrant workers’ return-to-hometown entrepreneurship. Section 3 presents the empirical design. Section 4 describes the data sources, variable definitions, and summary statistics. Section 5 reports the benchmark result, a series of robustness tests, heterogeneous results, and moderating effects. Section 6 offers a discussion of our findings. Section 7 are conclusion and policy implications.

## 2 Institutional background

### 2.1 IMIS

The IMIS in China aims to rectify disparities in healthcare coverage between urban and rural areas, with the objective of enhancing the equity and sustainability of universal healthcare coverage. In the past, this system encountered several significant challenges. First, urban residents enjoyed more comprehensive medical insurance coverage and higher reimbursement rates compared to their rural counterparts (38, 39). Second, there was a lack of uniformity between urban and rural medical insurance systems, resulting in substantial disparities in healthcare benefits (40). Third, the funding sources for rural residents’ medical insurance were unstable and often faced financial shortfalls (41, 42). To address these issues, the Chinese government initiated the process of implementing the IMIS. In 2003, the National Health and Family Planning Commission introduced the policy of establishing a multi-level medical security mechanism that covered both urban and rural areas (43). On January 12, 2016, the State Council issued the “Opinions on Integrating the Basic Medical Insurance System for Urban and Rural Residents,” announcing large-scale efforts to standardize urban and rural medical insurance systems nationwide.^1^ Subsequently, various provinces introduced corresponding implementation rules and policy measures to facilitate the rollout of the IMIS. Specific implementation timelines for each province (or municipality) are outlined in Table 1.

**Table 1:** The IMIS implementation in provinces (or municipalities).

Provinces (or municipalities)	Year
Tianjin, Chongqing	2009
Ningxia	2010
Guangdong	2012
Shandong	2014
Shanghai	2015
Fujian, Zhejiang, Hainan	2016
Yunnan, Inner Mongolia, Jilin, Sichuan, Anhui, Shanxi, Guangxi, Jiangxi, Hebei, Henan, Hubei, Hunan, Qinghai, Gansu, Xinjiang, Shaanxi	2017
Heilongjiang, Beijing, Jiangsu, Guizhou	2018
Liaoning	2019
Tibet	2020

The data of IMIS implementation year, collected by authors, is derived from the official website of each provincial or municipal government.

After the implementation of the IMIS, the situation of residents in both rural areas and urban areas improved significantly, and healthcare coverage was significantly enhanced in different dimensions (44). Firstly, the reimbursement rate was raised. For instance, in Shanghai, the hospitalization reimbursement rate was increased to 70% after the implementation of IMIS in 2017, compared to the previous rates of 60 and 50% for URBMI and NRCMS, respectively. In Beijing, both outpatient and inpatient reimbursement rates increased by 5% in 2018.^2^ In Shanxi Province, after the 2017 IMIS, the average reimbursement rate for total hospitalization expenses within the medical insurance catalog saw a 15% increase.^3^ Secondly, the list of reimbursed drugs was expanded. For instance, in Hebei Province, the original NRCMS covered approximately 1,000 types of drugs, while URBMI covered around 2,400 types. After the 2017 integration, the URRBMI expanded to cover approximately 2,900 types of drugs.^4^ In Yunnan Province, following the 2017 IMIS, the number of reimbursed drugs for rural residents increased by 1,476 types, and urban residents witnessed an increase of 442 types. This expansion of the drug catalog was more substantial for rural residents.^5^ Thirdly, a greater number of designated medical institutions were included. For instance, in Beijing, the number of designated medical institutions expanded from over 800 under NRCMS and more than 2,000 under URBMI to over 3,000 in total. In Shanxi Province, the number of designated medical and pharmaceutical institutions grew from over 2,000 to over 7,000.

However, there are still issues of fairness in the integration of this system. On one hand, the tiered payment and benefits do not truly reflect fairness. For instance, in Zhejiang Province, the urban–rural medical insurance is designed with multiple tiers, associating low contributions with low benefits and high contributions with high benefits (45). On the other hand, there is a lack of fairness in the reimbursement for offsite medical treatments. Due to the inconsistency in the scope of medical institutions providing diagnosis, treatment, and reimbursement services, patients seeking treatment in different areas may face difficulties in reimbursement based on the variations in qualifications among medical institutions. This diminishes the efficiency of healthcare insurance services and exacerbates regional disparities. Furthermore, disparities between local healthcare insurance policies and reimbursement systems hinder the settlement of healthcare insurance for offsite treatments, leading to regional disparities that affect the convenience of medical treatment for the public and increase the complexity of overall management (46).

### 2.2 Migrant workers’ return-to-hometown entrepreneurship

The trend of return-to-hometown entrepreneurship among Chinese migrant workers is becoming increasingly evident. According to statistics, the number of people who return to rural hometown for entrepreneurship exceeded 8.5 million in 2019,^6^ and it is projected to surpass 15 million nationwide by 2025.^7^ This phenomenon can be attributed to two main factors. On the one hand, there is the “push” factor from urban areas. This is evident in three ways. Firstly, China’s unique household registration (*hukou*) policy has exacerbated social exclusion of migrant workers (47). Secondly, the escalating cost of living results in heightened socioeconomic pressure on them. Thirdly, the number of jobs suitable for migrant workers is limited (48, 49). On the other hand, there is the “pull” factor in rural areas. This is evident in two ways. Firstly, improvements in rural infrastructure, including living conditions, transportation networks, and connectivity, may attract rural residents to return to their hometowns for entrepreneurship (50). Secondly, the Chinese Government has consistently emphasized the promotion of entrepreneurship among migrant workers who return to their hometowns to encourage the return of labor forces. For example, in 2014, the government recommended facilitating the transfer of rural migrant workers’ employment to nearby areas and providing support for them to return to their hometowns to start their own businesses.^8^

The success of migrant workers’ return-to-hometown entrepreneurship is of significant importance for the government to formulate sound entrepreneurship and rural development policies. Previous studies have provided evidence that the entrepreneurship of migrant workers returning to their hometowns, as a manifestation of the return of rural labor, complements rural human capital and contributes to the advancement of rural economic development (51). Return-to-hometown entrepreneurs possess advantages in terms of economic, human, and social capital, enabling them to swiftly seize market opportunities and leverage the diverse resource endowments in their local areas. This helps effectively rectify China’s longstanding pattern of one-way flow of high-quality capital from rural to urban areas (52). Moreover, it plays a crucial role in driving rural industrial transformation, upgrading, reducing the urban–rural development gap, contributing to the integrated development of urban and rural areas, and employment promotion (53, 54).

In summary, there may be an important connection between IMIS and the phenomenon of migrant workers’ return-to-hometown entrepreneurship. With the implementation of IMIS, not only has it provided greater security and stability in terms of healthcare for migrant workers, but it has also promoted urban–rural integration on a broader scale, creating more favorable conditions for their return-to-hometown entrepreneurship. This integration represents not only a significant advancement in healthcare but also a key driver for advancing socioeconomic development in rural areas. By improving health insurance coverage, IMIS boosts the confidence of migrant workers in returning to their hometowns for entrepreneurship. Furthermore, the experiences and skills they have accumulated in urban areas can be applied in rural regions, promoting diversification and innovation in local economies. Therefore, in-depth research on the impact of IMIS on the entrepreneurship of migrant workers returning to their hometowns not only holds theoretical significance but also has important practical value for guiding policy practices and promoting balanced urban–rural development.

## 3 Empirical strategies

### 3.1 Benchmark model

The traditional difference-in-differences (DID) model is applicable only when the timing of policy implementation is consistent. Given the uneven implementation timing of IMIS across various regions, this study follows the approach outlined by Beck et al. (55) and establishes a time-varying DID model to evaluate the impact of IMIS on migrant workers’ return-to-hometown entrepreneurship. The specification of the time-varying DID model is as follows:


(1) $$RE_{ipt}=\beta_{0}+\beta_{1}IMIS_{ipt}+\gamma X_{ipt}+\tau_{p}+\omega_{t}+\varepsilon_{ipt}$$

where $RE_{ipt}$ is the outcome variable, indicating whether the rural migrant worker i in the province p return to their hometowns to start an entrepreneurship in year t . $IMIS_{ipt}$ is the URRBMI integration conditions, which indicates whether IMIS was implemented in year t in the province p where individual i was located. $\beta_{1}$ is the coefficient of interest, which measures the causal impact of IMIS on return-to-hometown entrepreneurship among migrant workers. $X_{ipt}$ is a list of individual-level and household-level controls. $\tau_{p}$ denotes province fixed effects, which absorbs the impact of time-invariant province characteristics. $\omega_{t}$ denotes year fixed effects, which captures changes over time that affect respondents in different provinces similarly. $\varepsilon_{ipt}$ is the error term.

## 4 Data, variables and summary statistic

### 4.1 Data

The micro-level data for this study are sourced from the China Household Finance Survey (CHFS) conducted in four waves in the years 2013, 2015, 2017, and 2019. Initiated by the China Household Finance Survey and Research Center at Southwestern University of Finance and Economics in 2011, the CHFS is conducted biennially and has been updated to include data up to 2019. The most recent survey covers a sample distribution across 29 provinces (autonomous regions or municipalities directly under the central government), 367 counties (districts or county-level cities), and 1,481 communities, encompassing 40,011 households and 127,012 individuals. This survey collects data at three hierarchical levels: individual, household, and regional. It includes comprehensive information on population characteristics, employment status, mobility, housing assets, financial wealth, income, consumption, social security, insurance, and other relevant factors, rendering it well-suited for the purposes of our study. This wealth of information in the questionnaire enables us to select a multitude of control variables, thereby minimizing the potential for endogeneity bias due to omitted variables. Additionally, the four waves of longitudinal survey data align precisely with the primary implementation period of the IMIS.

We perform the following data preprocessing steps: Firstly, as the implementation of IMIS primarily affected individuals with agricultural *hukou*, and return-to-hometown entrepreneurship was most prevalent among the working-age population, we filter the CHFS database to include only individuals with agricultural *hukou* aged between 18 and 65. Secondly, in the 2017 survey wave of the CHFS database, the question used to assess residents’ “risk preferences” was posed exclusively to new households, leading to missing values for this variable in the tracking sample. To address this, we impute missing values using responses from the original households in 2015.^9^ Thirdly, the timing of IMIS implementation varies among different prefecture-level cities within individual provinces. Since the micro-level database only allows for province-level identification, we exclude provinces where there is inconsistency in policy implementation timing among prefecture-level cities within the same province. In the end, we have a total of 50,559 valid samples.

### 4.2 Definitions of variables

**Outcome variable**: the outcome variable is *Return-to-hometown entrepreneurship* (RE), which is a dummy variable that equals 1 if an individual has returned to their hometown and is involved in work categorized as “self-employed, operating an individual or private business, or running an online store”^10^ during the survey year, and 0 if otherwise.

**Independent variables**: the independent variable is *IMIS*. We determine the implementation time of IMIS in various prefecture-level cities based on detailed implementation documents. It is a dummy variable which equals 1 if the individual’s respondence takes place before the implementation of IMIS in his/her province, and 0 if otherwise.

**Control variables**: given the high relevance of individual and household-level factors to migrant workers’ decisions to engage in entrepreneurship upon returning to their hometowns, following Shi et al. (56), Guo and Wang (57), and Chen and Zhang (58), we incorporate control variables at individual and household levels. Specifically, individual-level variables include *Age*, *Age^2^*, *Gender*, *Education level*, *Marital status*, *Annual income*, *Self-reported health*, *Pension insurance*, *Medical insurance*, and *Risk preference. Age* is defined as the difference between the individual’s survey wave and year of birth. Considering the nonlinear effect of age on migrant workers’ return-to-hometown entrepreneurship, we include a squared term for age, i.e., *Age*.^2^ *Gender* is a dummy variable which equals 1 if the individual is male, and 0 if otherwise. *Educational level* is defined on a corresponding scale from 0 to 8, with values assigned based on the individual’s highest reported educational level, ranging from illiteracy (0) to primary school (1), junior high school (2), senior high school (3), vocational school (4), associate’s degrees (5), bachelor’s degrees (6), master’s degrees (7), and doctoral degrees (8).^11^ *Marital status* is a dummy variable which equals 1 if the individual is married, cohabiting, or remarried, and 0 if otherwise. *Annual income* is the logarithm of all the income an individual earns in a year, including salary, bonuses, money management, etc. Based on an individual’s response to the question, “Compared to people of your age, how would you rate your current physical health?,” *Self-reported health* is a dummy variable which equals 0 if the response is poor or very poor, and 1 if otherwise. *Social pension insurance* is a dummy variable which equals 1 if the individual has any form of pension insurance,^12^ and 0 if otherwise. *Social medical insurance* is a dummy variable which equals 1 if the individual has any form of medical insurance,^13^ and 0 if otherwise. Based on the response to the question, “If you have a sum of money for investment, which type of investment project would you prefer?,” *Risk preference* takes a value of 0 for not willing to take any risks, 1 for slightly low risk, 2 for moderate risk, 3 for slightly high risk, and 4 for high risk. Household-level variables include *Family size*, *Number of members under 18*, and *Household renqing*^14^ *expenditures. Family size* refers to the number of family members economically connected to the household. *Number of members under 18* is the number of individuals in the household who are younger than 18 years old. *Household renqing expenditures* represent the logarithm of cash or non-cash expenditures made by the household to individuals other than the parents of both spouses.

### 4.3 Summary statistics

Table 2 presents sample characteristics based on the implementation of IMIS in the regions where individuals reside. It includes mean and standard deviation values for the full sample, the IMIS subsample, and the non-IMIS subsample, along with significance tests for differences in means between these two subsamples. The results in Table 2 reveal that from 2013 to 2019, the proportion of individuals engaged in return-to-hometown entrepreneurship among the working-age population remained relatively low, accounting for only 1.18%. Moreover, the IMIS subsample shows significantly higher levels of individual annual income and subjective well-being compared to the non-IMIS subsample. Additionally, the statistical results for various control variables suggest that individuals in the IMIS subsample, when compared to those in the non-IMIS subsample, exhibit characteristics such as older ages, higher educational attainment, lower marriage rates, higher income levels, better health status, a higher participation rate in social pension insurance, a lower participation rate in social medical insurance, lower risk preferences, fewer family members, and lower family *renqing* expenditures.

**Table 2:** Summary statistics.

Variables	Total sample	IMIS subsample	Non-IMIS subsample	Difference
Panel A: Outcome variables				
Return-to-hometown entrepreneurship	0.0118 (0.0004)	0.0168 (0.0007)	0.0048 (0.0004)	0.0120^***^ (0.0009)
Panel B: Individual variables				
Age	39.9053 (0.0448)	40.2928 (0.0594)	39.3594 (0.0678)	0.9334^***^ (0.0908)
Gender	0.6291 (0.0019)	0.6269 (0.0025)	0.6322 (0.0029)	−0.0053 (0.0038)
Educational level	2.3440 (0.0054)	2.3847 (0.0072)	2.2868 (0.0083)	0.0978^***^ (0.0110)
Marital status	0.8247 (0.0015)	0.8138 (0.0020)	0.8401 (0.0022)	−0.0263^***^ (0.0030)
Annual income	10.8326 (0.0054)	10.9543 (0.0064)	10.6613 (0.0093)	0.2929^***^ (0.0109)
Self-reported health	0.0830 (0.0011)	0.0865 (0.0014)	0.0780 (0.0016)	0.0085^***^ (0.0022)
Social pension insurance	0.6406 (0.0019)	0.6529 (0.0024)	0.6232 (0.0029)	0.0297^***^ (0.0038)
Social medical insurance	0.9299 (0.0010)	0.9265 (0.0013)	0.9346 (0.0015)	−0.0080^***^ (0.0020)
Risk preference	0.8202 (0.0044)	0.7742 (0.0056)	0.8850 (0.0071)	−0.1107^***^ (0.0090)
Panel C: Household-level variables				
Family size	4.2116 (0.1327)	4.1565 (0.0085)	4.2892 (0.0103)	−0.1327^***^ (0.0133)
Number of members under 18	0.8297 (0.0035)	0.8281 (0.0047)	0.8319 (0.0053)	−0.0038 (0.0072)
Household *renqing* expenditures	5.8288 (0.0139)	5.4597 (0.0189)	6.3487 (0.0200)	−0.8890^***^ (0.0280)

Standard errors are shown in parentheses. ***, ** and * indicate the t-test significance levels of the difference in mean values between IMIS and non-IMIS subsamples. The significance levels of 1, 5, and 10% are denoted by ***, **, and *, respectively.

## 5 Results

### 5.1 Common trends test

The main advantage of the time-varying DID identification strategy is its ability to control for unobserved confounders that change over time, which could otherwise bias our estimates. However, its validity relies on the parallel trends assumption, which posits that in the presence of time-fixed effects, province-fixed effects, and individual and household-level control factors, there should be no significant differences in the changes of the outcome variable across provinces prior to the policy shock. In other words, any potential confounders that are not directly observed in the data would follow a parallel path in both the treatment and control groups in the absence of the policy intervention. By comparing the differences in outcomes before and after the policy implementation across these groups, we can isolate the impact of the policy from these unobserved factors. Furthermore, this method enhances the credibility of our findings by allowing for a more nuanced analysis of the policy’s impact. It enables us to observe how the effect of the policy unfolds over time, which is crucial in understanding the dynamics of policy implementation and its consequences.

The event study approach is employed in various ways to assess the impact of policies on outcome variables. First, it enables an examination of dynamic changes in outcome variables before and after policy implementation by defining relative time windows. Analyzing the pre-policy change trend helps ascertain whether the parallel trends assumption holds, while observing the post-policy dynamic trend provides insights into the policy’s long-term effects. Second, the event study method contributes to more robust causal identification by accounting for a range of observable and unobservable confounding factors, thus enhancing the effectiveness of causal inference. Consequently, we estimate the following event study specification:


(2) $$RE_{ipj}=\beta_{0}+\overset{3}{\underset{j=-5}{\sum }}\beta_{1}IMIS_{pj}+\gamma X_{ipj}+\tau_{p}+\omega_{j}+\varepsilon_{ipj}$$

where the subscript *j* is the relative period between the time when individual *i* receives the interview and the implementation of IMIS in his/her province. For instance, j = 0 means that IMIS has been implemented in the current year in the province where individual i is located. The settings for other variables are the same as those in Equation (1).

**Figure 1:**
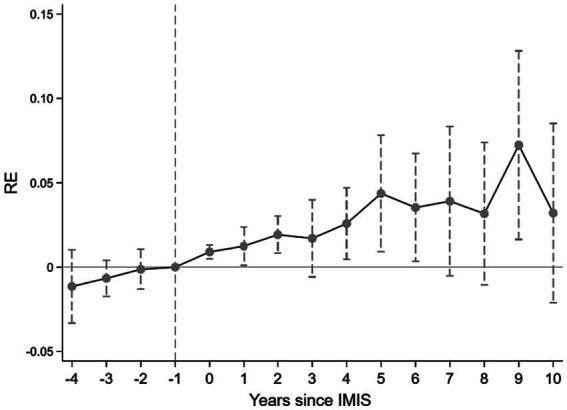
Event study results.

Figure 1, based on Equation (2), presents the annual trend for RE. We utilize the year preceding IMIS implementation in each province as the reference point. Prior to IMIS, the coefficients of the interaction terms exhibit small absolute magnitudes and statistical insignificance. This suggests that there is no significant difference in the change of the outcome variable between provinces before the implementation of IMIS. After IMIS, the 95% confidence intervals (CIs) of the interaction term coefficients overlap, indicating that the common trends assumption holds for the outcome variable, namely, RE.

### 5.2 Benchmark results

Table 3 presents the benchmark results based on Equation (1). Specifically, in column (1), no control variables are included. Column (2) introduces individual-level control variables, while column (3) adds household-level control variables to those in column (2). Column (4) further includes time fixed effects and province fixed effects. The results demonstrate that the implementation of IMIS significantly enhances the likelihood of migrant workers returning to their hometowns for entrepreneurial endeavors. The magnitude of this effect ranges from 0.44 to 1.34%, and all estimated coefficients are statistically significant at the 1% level. We consider the results from column (4) as the benchmark, indicating that IMIS significantly increases the probability of migrant workers returning to their hometowns to start a business by 0.44%. This unintended finding holds significant practical implications for China, especially in the context of the ongoing implementation of the Rural Revitalization Strategy,^15^ as China has long grappled with issues related to rural labor outflow and shortages (59). The reflow of labor is expected to play a pivotal role in improving the distribution of labor between urban and rural areas, boosting rural economic vitality, narrowing the urban–rural gap, and establishing a unified national labor market (60).

**Table 3:** Benchmark results.

Variable	(1)	(2)	(3)	(4)	(5)
IMIS	0.0120***	0.0128***	0.0134***	0.0044***	0.0295***
	(0.001)	(0.001)	(0.001)	(0.002)	(0.002)
Age		0.0024***	0.0022***	0.0021***	0.0024***
		(0.000)	(0.000)	(0.000)	(0.000)
Age2		−0.0000***	−0.0000***	−0.0000***	−0.0000***
		(0.000)	(0.000)	(0.000)	(0.000)
Gender		0.0031***	0.0031***	0.0031***	0.0041***
		(0.001)	(0.001)	(0.001)	(0.001)
Education level		0.0011***	0.0010***	0.0009***	0.0011**
		(0.000)	(0.000)	(0.000)	(0.001)
Marital status		0.0058***	0.0031***	0.0050***	0.0051***
		(0.001)	(0.001)	(0.001)	(0.002)
Self-reported health		−0.0031**	−0.0026*	−0.0033**	−0.0031
		(0.001)	(0.001)	(0.001)	(0.002)
Social pension insurance		−0.0024***	−0.0027***	−0.0022**	−0.0022
		(0.001)	(0.001)	(0.001)	(0.001)
Social medical insurance		0.0050***	0.0047***	0.0042***	0.0034
		(0.001)	(0.001)	(0.001)	(0.003)
Annual income		−0.0015***	−0.0016***	−0.0021***	−0.0028***
		(0.000)	(0.000)	(0.000)	(0.001)
Family size			−0.0008***	−0.0009***	−0.0016***
			(0.000)	(0.000)	(0.001)
Number of members under 18			0.0037***	0.0031***	0.0045***
			(0.001)	(0.001)	(0.001)
Household *renqing* expenditures			0.0008***	0.0010***	0.0011***
			(0.000)	(0.000)	(0.000)
Risk preference			0.0013***	0.0020***	0.0027***
			(0.000)	(0.000)	(0.001)
Constant	0.0048***	−0.0399***	−0.0390***	−0.0436***	−0.0302***
	(0.000)	(0.007)	(0.007)	(0.007)	(0.011)
Time fixed effects Province fixed effects				YES YES	YES YES
Observations	65,770	65,770	65,770	65,770	32,769
*R*-squared	0.003	0.006	0.007	0.015	0.017

Standard errors are clustered at the individual level. ***, **, and * denote significance levels of 1, 5, and 10%, respectively.

### 5.3 Robustness checks

#### 5.3.1 DIDM

Following the principles of staggered DID, and considering the varying timing of IMIS implementation across different regions, we are concerned that some groups that have already experienced the treatment may inadvertently be used as control groups in the traditional DID approach. This can result in the problem of negative weights, potentially biasing the policy effect (61). To investigate this hypothesis, we adopt the approach suggested by de Chaisemartin and D’Haultfoeuille (62) to address the negative weights issue, particularly when the Average Treatment Effects (ATEs) exhibit heterogeneity across groups or time periods. The method focuses on correctly identifying and employing control and treatment groups in the context of staggered policy implementation. It accounts for the heterogeneity in ATEs across different groups and time periods, which is a common occurrence in staggered policy settings. Specifically, we employ a DID_g,t_ estimator to assess the impact of IMIS on migrant workers’ return-to-hometown entrepreneurship. By using a DID_g,t_ estimator, this approach allows for a more accurate assessment of the policy’s impact, factoring in the varying timings of its implementation and the diverse effects it may have across different regions or groups. The DID_g,t_ estimation is specified as Equation (3):

(3) $$DID_{g,t}=E\left(Y_{g,F_{g}+l}\left(0_{F_{g}-1},1_{l+1}\right)-Y_{g,F_{g}+l}\left(0_{F_{g}+l}\right)\right)$$

where DID_g,l_ is the expected difference between the treated individual $g^{\prime }s$ status about whether they return to their hometowns to start an entrepreneurship at $F_{g}+l$ (denoted by $Y_{g,F_{g}+l}\left(0_{F_{g}-1},1_{l+1}\right)$ ) and the counterfactual “status quo” status for an individual would have obtained if an individual had remained untreated from period one to $F_{g}+l$ (denoted by $Y_{g,F_{g}+l}\left(0_{F_{g}+l}\right)$ ). $Y_{g,F_{g}+l}$ denotes an individual $g^{\prime }s$ statues about whether they return to their hometowns to start an entrepreneurship at period $F_{g}+l$ ,and the treatment condition from period one to $F_{g}+l$ are defined in the brackets. $F_{g}$ denotes the first period at which individual g’s IMIS was implemented. l is the length of exposure to the treatment. $0_{F_{g}-1}$ and $0_{F_{g}+l}$ denote $1\times \left(F_{g}-1\right)$ and $1\times \left(F_{g}+l\right)$ vectors with coordinates equal to 0. Similarly, $1_{l+1}$ denote a 1×(l+1) vector with coordinates equal to 1. $DID_{g,l}$ estimators are aggregated into a $DID_{l}$ estimator, to estimate the effect of IMIS on individuals’ status about whether they return to their hometowns to start an entrepreneurship for l periods. As recommended by de Chaisemartin and D’Haultfœuille (62), this study clusters the standard errors to the individual level.

de Chaisemartin and D’Haultfœuille (62) shows $DID_{l}$ estimator can be extended to conditional DID designs and is unbiased under conditional parallel trends assumption which requires request the migrant workers’ return-to-hometown entrepreneurship in the region before the occurrence of the IMIS has the same trend. To test the parallel trends assumption, the placebo estimators are used corresponding to the ATEs estimated before the implementation of the IMIS. The $DID_{l}$ and placebo estimators are computed by the did_multiplegt package for Stata (63).

**Figure 2:**
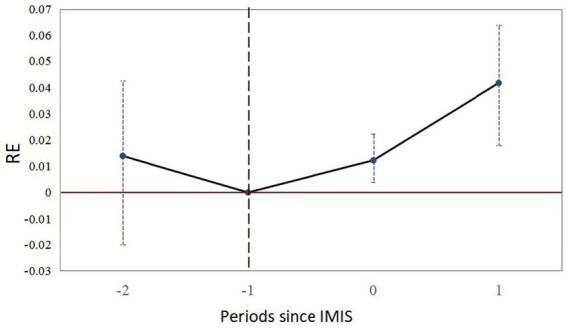
Dynamic effects.

Columns (1)–(3) of Table 4 present the results of the impact of IMIS on RE among migrant workers using the de Chaisemartin and D’Haultfœuille (62) approach. In column (1), without the inclusion of control variables, the estimated coefficient for IMIS stands at 0.0126, and it is statistically significant (*p* < 0.01). When we control for individual and household characteristics in column (2), the coefficient decreases to 0.0093 but remains statistically significant (*p* < 0.01). Notably, the results in columns (1) and (2) capture the transient effect of IMIS, representing the immediate impact of the policy. To provide a more precise estimate of the policy effect, accounting for the post-policy impact, we estimate the dynamic effect in column (3) and calculate the average policy effect of IMIS within our study waves. In column (3), the first and second rows of results suggest that the effect of IMIS in promoting migrant workers’ return-to-hometown entrepreneurship gradually increases over time, consistent with the post-policy trend in Figure 1. The third row in column (3) represents the average effect of IMIS, while the fourth row demonstrates that all results of the placebo test in the pre-policy period, as seen in columns (1)–(3), are not statistically significant, aligning with the pre-policy trend in Figure 1. Figure 2 visually illustrates the dynamic trends observed in column (3) of Table 4. This outcome implies that the policy effects resulting from IMIS implementation are not incidental, substantiating their significance.

**Table 4:** The impact of IMIS on RE among migrant workers using DIDM.

Variable	(1) DIDM	(2) DIDM	(3) DIDM
IMIS_t_	0.0126^***^	0.0093^***^	0.0079^***^
	(0.0036)	(0.0032)	(0.0028)
IMIS_t + 1_			0.0422^***^
			(0.0068)
Average			0.0110^***^
			(0.0027)
Placebo_1	−0.0139	−0.0156	0.0127
	(0.0162)	(0.0106)	(0.0095)
Control variables	No	Yes	Yes
Observations	65,770	65,770	65,770

Control variables are included at individual and household levels as shown in Table 3. Standard errors are clustered at the individual level. ***, **, and * denote significance levels of 1, 5, and 10%, respectively.

#### 5.3.2 Endogeneity

As presented in Table 1, the implementation of the IMIS across provinces exhibits a staggered pattern. It is essential to recognize that the timing of IMIS implementation in specific provinces may not have been a random occurrence but rather a result of policy considerations stemming from endogenous factors, including distinct local economic, demographic, and environmental conditions.

This raises doubts about the credibility of the baseline regression results in our study. China’s work plan for IMIS mandates that each region should formulate specific implementation plans by the end of December 2016 and put them into action promptly. Consequently, when comparing cities that implemented IMIS in the vicinity of 2017 with those that did so in the year 2017, it becomes evident that the latter group was more likely to be influenced by the exogenous deadline stipulated by national policy. In other words, the provinces that implemented IMIS in 2017 are more conducive to aiding us in mitigating endogeneity concerns arising from autonomous decisions. In this section, we confine our analysis to provinces that adopted IMIS in the year 2017 and proceed to re-evaluate the policy impacts of IMIS in accordance with Equation (1). The result in column (5) of Table 3 indicates that the coefficient for IMIS remains significantly positive at the 1% level of significance, which substantially alleviates concerns about endogeneity issues in our findings.

#### 5.3.3 Alternative model

In the benchmark model, we utilize OLS regression. However, since the outcome variable in this study, i.e., RE, is binary, and recognizing that different model specifications can potentially impact the results, in this section, we conduct Probit and Logit regressions on Equation (1). Probit^16^ and Logit^17^ models are both used in statistics for binary outcome modeling, where the response variable can take one of two possible outcomes. These models are particularly useful in situations where the outcome is a probability of an event occurring. The results of marginal effects presented in Table 5 demonstrate that the outcomes, following the model replacement, do not significantly deviate from the benchmark results. This indicates that our core findings remain robust across various model specifications.

**Table 5:** Alternative model.

Variable	(1)	(2)
	Probit	Logit
IMIS	0.0048**	0.0050**
	(0.0020)	(0.0022)
Control variables	YES	YES
Time fixed effects Province fixed effects	YES YES	YES YES
Observations *R*-squared	65,770 0.1147	65,770 0.1162

Control variables are included at individual and household levels as shown in Table 3. Standard errors are clustered at the individual level. ***, **, and * denote significance levels of 1, 5, and 10%, respectively.

#### 5.3.4 Placebo test

Given the possibility that changes in trends for the treatment and control groups after IMIS implementation may be influenced by other policies or random factors, it becomes imperative to conduct a placebo test to ascertain whether IMIS was indeed the driving factor, thereby eliminating the potential contamination of results by other unobservable factors. In this section, we undertake a placebo test by randomizing the treatment and control groups. The specific procedure is as follows: Firstly, we maintain the original policy implementation timing unchanged. If n regions initiate IMIS implementation in year t, we randomly select n regions from all regions as those where IMIS commences in that year. At this stage, these n regions form the newly generated random treatment groups, while the remaining regions naturally become the newly generated random control groups. Subsequently, based on this assignment, we re-estimate Equation (1) using the newly generated random treatment and control groups and repeat this process 500 times. This results in 500 estimated coefficients for IMIS implementation.

**Figure 3:**
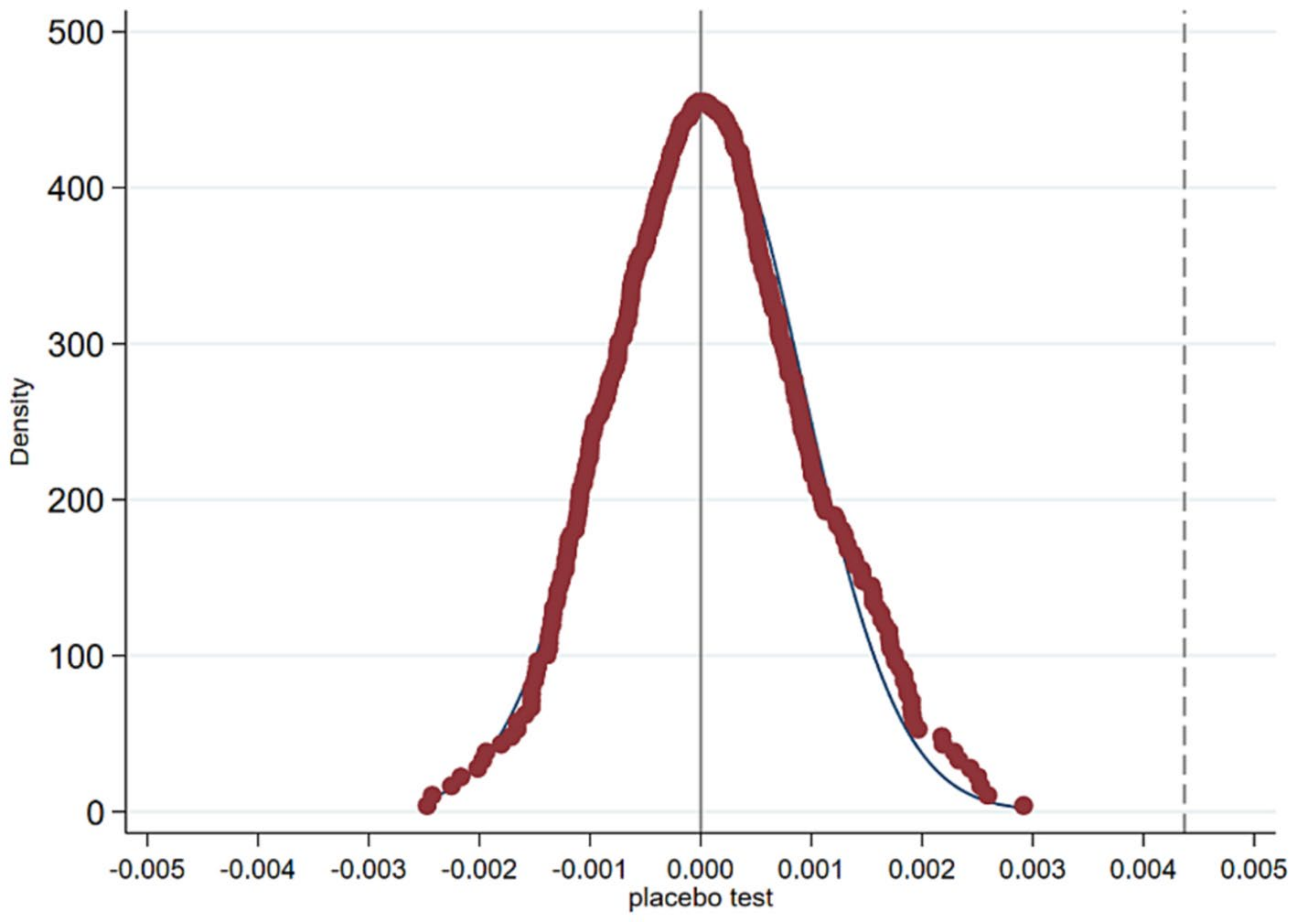
Placebo test.

The probability density distribution of the estimated coefficients from randomization is depicted in Figure 3. The results reveal that the distribution of estimated coefficients for IMIS closely approximates a normal distribution with a mean near 0. When we compare this distribution with the coefficient of 0.0044 for IMIS implementation in column (4) of Table 3, it becomes evident that the coefficient in the baseline regression significantly differs from the coefficient in the placebo test. As a result, we can conclude that our baseline finding, indicating that IMIS promotes return-to-hometown entrepreneurship among rural migrant workers, is less likely to be influenced by other policies or unobservable random factors.

### 5.4 Heterogeneity analysis

**Gender**: within Panel A of Table 6, we furnish estimates stratified by gender. Findings suggest a positive yet statistically insignificant impact of IMIS on female migrant workers. In contrast, male migrant workers exhibit a more pronounced inclination to return to their hometowns for entrepreneurial endeavors, evidenced by a statistically significant effect of 0.0051 (*p* < 0.05). This disparity implies the presence of gender-based variations in the effectiveness of IMIS in fostering entrepreneurship that draws individuals back to their hometowns among migrant laborers.

**Table 6:** Heterogeneity results.

Variables	(1)	(2)
Panel A: Subgroups by gender		
	Female	Male
IMIS	0.0030	0.0051^**^
	(0.0025)	(0.0021)
Observations	24,395	41,375
Panel B: Subgroups by age		
	≤45	>45
IMIS	0.0060^***^	0.0025
	(0.0020)	(0.0030)
Observations	41,020	24,750
Panel C: Subgroups by educational level		
	Low	High
IMIS	0.0050^**^	0.0026
	(0.0020)	(0.0029)
Observations	46,189	19,581
Panel D: Subgroups by annual income		
	Low	High
IMIS	−0.0015	0.0088^***^
	(0.0027)	(0.0020)
Observations	32,885	32,885
Panel E: Subgroups by risk preferences		
	Low	High
IMIS	0.0061^***^	0.0005
	(0.0018)	(0.0034)
Observations	48,299	17,471
Panel F: Subgroups by social networks		
	Small	Large
IMIS	0.0033^**^	0.0057^**^
	(0.0020)	(0.0026)
Observations	32,885	32,885
Control variables	Yes	Yes

Control variables are the same as in Table 3. Standard errors are clustered at the individual level. ***, **, and * denote significance levels of 1, 5, and 10%, respectively.

**Age**: according to the age classification criteria for middle-aged and older adults established by the World Health Organization (64–66), we divided the total sample into two sub-samples based on the median age of 45. As demonstrated in Panel B of Table 6, IMIS enhances the likelihood of migrant workers under 45 returning to their hometowns for entrepreneurial endeavors by 0.60% (*p* < 0.01). However, among migrant workers over the age of 45, IMIS does not have a significant effect on their return to business. This indicates that IMIS has a relatively stronger influence on encouraging younger individuals to return to their hometowns for entrepreneurial activities.^18^

**Education Level**: we divide the total sample into two sub-samples based on educational levels, distinguishing between low and high education. Specifically, rural migrant workers with education levels of ‘illiteracy,’ ‘primary school,’ and ‘junior high school’ were classified as having low education, while others were categorized as having high education.^19^ Findings in Panel C of Table 6 reveal that, for migrant workers with lower education, IMIS significantly increases their probability of returning for entrepreneurial activities by 0.50% (*p* < 0.05). Conversely, for those with higher education, the effect of IMIS is positive but not statistically significant. This suggests that migrant workers with comparatively lower educational attainment are more inclined to return to their hometowns for entrepreneurship following the implementation of IMIS, consistent with the findings of Tamvada et al. (67).

**Annual income**: the total sample was divided based on median income, distinguishing between low-income (<median) and high-income (≥median) migrant workers. The findings in Panel D of Table 6 reveal that IMIS significantly enhances the likelihood of return-to-hometown entrepreneurship for high-income migrant workers by 0.88% (*p* < 0.01). However, this effect is not statistically significant for low-income migrant workers. This outcome implies that IMIS exerts a more substantial incentive impact on migrant workers with relatively higher annual incomes compared to those with lower annual incomes.

**Risk preferences**: in Panel E of Table 6, estimates based on individuals’ risk preferences are outlined. The total sample was divided into two sub-samples: one with lower risk preferences and another with higher risk preferences. The findings show that IMIS significantly raises the probability of return-to-hometown entrepreneurship by 0.61% (*p* < 0.01) for those with lower risk preferences. Conversely, the effect of IMIS on individuals with higher risk preferences, while positive, fails to attain statistical significance. This implies that IMIS exerts a more substantial incentive effect on migrant workers with lower risk preferences compared to those with higher risk preferences.

**Social networks**: the findings presented in Panel F of Table 6 demonstrate that the impact of IMIS on return-to-hometown entrepreneurship is contingent upon the magnitude of migrant workers’ social networks, assessed through *renqing* expenditures.^20^ Specifically, migrant workers with larger social networks are significantly more inclined to return to their hometowns for entrepreneurship following IMIS, with a significant increase of 0.57% (*p* < 0.05). On the other hand, the influence of IMIS on those with smaller social networks is less conspicuous, registering a change of 0.0033, which is not statistically significant—a circumstance potentially attributed to the smaller sample size. These results indicate that IMIS exerts a more potent motivating effect on migrant workers with expansive social networks compared to those with more limited networks.

### 5.5 Moderating effect of MEI

In 2015, “Mass Entrepreneurship and Innovation”(MEI) was incorporated into the Chinese government’s work report. Subsequently, in 2016, China issued several policy documents aimed at promoting innovation and entrepreneurship among its residents. Therefore, the implementation of MEI occurred within the study period of this paper. A natural question arises: will the primary effect of IMIS in promoting return-to-hometown entrepreneurship among migrant workers be affected by MEI? To control for the potential contamination effects of MEI and examine how MEI might impact our results, we introduce the implementation year of MEI as a moderating variable and establish the following moderation effect model:


(4) $$\begin{array}{c} RE_{ipt}=\beta_{0}+\beta_{1}IMIS_{ipt}+\beta_{2}MEI+\beta_{3}IMIS_{ipt}\times MEI\\ +\;\gamma X_{ipt}+\tau_{p}+\omega_{t}+\varepsilon_{ipt} \end{array}$$

where the MEI is the moderator variable, a dummy variable which takes the value of 1 if the survey waves occurred in 2013 and 2015, and takes the value of 0 if the survey waves occurred in 2017 and 2019. $\beta_{1}$ represents the impact of IMIS on migrant workers’ return-to-hometown entrepreneurship, controlling for the influence of MEI. $\beta_{2}$ signifies the influence of MEI on migrant workers’ return-to-hometown entrepreneurship, controlling for the effect of IMIS. $\beta_{3}$ denotes the interactive effect between IMIS and MEI on migrant workers’ return-to-hometown entrepreneurship. In other words, it elucidates how MEI moderates the impact of IMIS on migrant workers’ return-to-hometown entrepreneurship. The settings for other variables are the same as those in Equation (1).

In Table 7, based on Equation (4), the moderating effect of MEI is analyzed. Column (3) indicates a positive but non-significant coefficient for IMIS (*p* > 0.1), suggesting that the impact of the IMIS on migrant workers’ entrepreneurship becomes less significant when considering MEI. Conversely, the coefficient of MEI is significant (*p* < 0.05), underscoring a stronger influence of MEI on entrepreneurship under the control of IMIS. Notably, the interaction between IMIS and MEI shows an increased coefficient of 0.0069 (*p* < 0.01), compared to IMIS’s coefficient of 0.0044 (*p* < 0.01) in Column (4) of Table 3. This implies that MEI amplifies the effect of IMIS in encouraging migrant workers to start businesses in their hometowns. The result is unsurprising, as IMIS meets the basic healthcare needs of migrant workers, providing a more stable medical safeguard, thereby reducing entrepreneurial risks. Concurrently, the MEI policy offers substantial entrepreneurial opportunities and support. These two policies likely create synergistic and complementary effects in different aspects, fostering a more conducive environment for entrepreneurship. This invigorates migrant workers’ enthusiasm for returning to their hometowns to start businesses.

**Table 7:** Moderating effect of MEI.

Variables	(1)	(2)	(3)
IMIS	−0.0016 (0.0015)	−0.0010 (0.0015)	0.0017 (0.0022)
MEI	0.0014 (0.0014)	0.0043*** (0.0015)	0.0035** (0.0018)
IMIS×MEI	0.0158*** (0.0005)	0.0182*** (0.0012)	0.0069*** (0.0013)
Control variables	No	Yes	Yes
Province fixed effects	No	No	Yes
Observations	65,770	65,770	65,770
*R* ^2^	0.0040	0.0086	0.0122

Control variables are included at individual and household levels as shown in Table 3. Standard errors are clustered at the individual level. ***, **, and * denote significance levels of 1, 5, and 10%, respectively.

## 6 Discussion

The primary result reveals that IMIS significantly increases the probability of migrant workers engaging in return-to-hometown entrepreneurship by 0.44%, which still holds after a series of robustness tests. According to the “Statistical Communique of the People’s Republic of China on National Economic and Social Development for the Year 2022,”^21^ in 2022 there were approximately 171.9 million migrant workers in China. According to our calculations, IMIS has the potential to facilitate the return of 756,000 individuals to rural areas for entrepreneurial pursuits. It is essential to note that this is a relatively conservative estimate, as it may be affected by bias due to the presence of negative weights in the DID estimation. Upon addressing this issue, the magnitude of the effect could potentially escalate to around 1%. This increase can be attributed to the improvements in welfare benefits compared to the previous NCRMS, particularly the expansion of medical insurance generosity and higher reimbursement rates. With the reduction in disparities between urban and rural medical coverage, some migrant workers may be more inclined to return to rural areas due to lower living costs, both for residence and entrepreneurship, thereby contributing to the return of the original rural workforce (68). While prior research has not specifically assessed the effect of IMIS on the return-to-hometown entrepreneurship of migrant workers, certain studies have explored the influence of health insurance on the employment, entrepreneurship or pullback effect of migrant workers (3, 68, 69), and their findings are consistent with this paper.

The results of the heterogeneity show that IMIS has a higher intensity of impact on male migrant workers returning to their hometowns for entrepreneurial endeavors. Possible explanations for this divergence include, firstly, within traditional Chinese cultural norms, males typically shoulder the role of primary breadwinners, whereas women are predominantly tasked with family care responsibilities. This dynamic may pose additional challenges for women contemplating a return to entrepreneurship in their hometowns (3). Secondly, gender inequalities in the labor market, which may result in greater challenges for females in accessing necessary resources for entrepreneurship, including funding, technical support, and networks (70). Thirdly, gender differences in industry or professional preferences in the context of hometown entrepreneurial ventures may also contribute to their varied responses to IMIS (71). For instance, females may lean toward social enterprises or community-oriented entrepreneurship, whereas males may focus more on commercial gains and expansion.

In terms of age, there is an increased probability of younger individuals engaging in hometown entrepreneurship after the implementation of IMIS. In the context of China’s rural labor shortage, particularly among the youth, this trend undoubtedly contributing to alleviating the issue of rural hollowing. The reasons for this phenomenon can be reasonably explained by the Life Cycle Theory and Human Capital Theory (72, 73). The former suggests that younger individuals possess greater flexibility in career choices and geographic mobility, while the latter emphasizes the investment of young people in education and skills, which may predispose them to capitalize on opportunities for returning to their hometowns to start businesses.

Concerning educational level, migrant workers with lower education levels typically have fewer savings and limited social security (74). These circumstances can make them more apprehensive about potential medical expenses, which in turn could pose a significant obstacle to their entrepreneurial aspirations when returning home. The implementation of IMIS has expanded healthcare coverage, providing them with the confidence required for participation in rural entrepreneurial activities, thus they may be more inclined to return to rural areas to seek or create job opportunities (44). Furthermore, migrant workers with higher incomes typically possess increased savings and financial resources, providing them with a financial buffer that enables the initiation and sustenance of entrepreneurial endeavors (75). The implementation of IMIS unlocks precautionary savings that can be redirected toward investments in entrepreneurship, significantly increasing their likelihood of returning home for business ventures.

We also creatively analyze heterogeneity in risk preferences and social networks. Due to the different risk preferences of individual migrant workers, the impact of IMIS on their return to hometowns to become an entrepreneur is not the same. One plausible explanation is that IMIS, by alleviating concerns regarding health risks and medical expenses (76), provides a degree of economic security (77). This observation is consistent with Risk Aversion Theory, positing that risk-averse individuals are inclined to favor options that minimize uncertainty and potential losses. For individuals with lower risk preferences, they may be more inclined to prioritize their health, economic security, and family well-being (22). As a result, they show more caution before making decisions (78), which lead to their hesitation to embrace the risks of entrepreneurship prior to IMIS, and in turn, they chose to return to their hometowns for entrepreneurship.

We have found that social networks are instrumental in entrepreneurship (79). This observation can be elucidated through the framework of social capital theory, emphasizing the crucial role of social networks in furnishing resources, information, and social support (80). Additionally, the size of one’s social network may potentially correlate with entrepreneurial motivation. For example, a larger network implies greater social support (capital, land, equipment, or other forms of support) and encouragement (relatives, friends, fellow townspeople, etc.), possibly leading to more opportunities and ideas for entrepreneurship. This, in turn, is vital for stimulating the entrepreneurial interest and enthusiasm of migrant workers, as well as for reducing the perception of entrepreneurial risks and enhancing confidence in their entrepreneurial pursuits (81). However, it is important to note that our proxy variable for social networks, *renqing* expenditures, may not fully encapsulate the complexities of these relationships. The formation and sustenance of social networks are multifaceted, involving elements like family ties, friendships, professional connections, and participation in social activities (82). *Renqing* expenditures represent just one dimension of these interactions, mainly indicative of the level of friendly interactions, cooperation, and mutual assistance. Consequently, while these results are enlightening, they ought to be interpreted cautiously due to the inherent limitations of using *renqing* expenditures as a surrogate for the breadth of social networks (83).

The results pertaining to the moderating effect reveal a crucial fact: while the IMIS exerts a positive impact on the entrepreneurial decisions of migrant workers, this influence is significantly amplified when combined with MEI. In other words, there exists a distinct synergistic effect between medical security and entrepreneurship support policies. This indicates that to effectively promote entrepreneurship among returning migrant workers, a solitary policy approach may be insufficient in yielding substantial outcomes, and the implementation of comprehensive policy measures is crucial. This conclusion aligns with established theoretical frameworks, suggesting that an integrated policy approach can more effectively stimulate and support the actions of the target group.

We acknowledge potential limitations in our study: Firstly, due to data limitations, we could only analyze information from four survey waves, limiting our ability to capture comprehensive pre- and post-trends. Nevertheless, we believe that our results still offer valuable insights and implications. Secondly, while we have presented empirical evidence of IMIS’s role in promoting the return of rural labor for entrepreneurship, the ultimate outcomes of migrant workers’ entrepreneurship remain uncertain, necessitating further exploration in future studies. Thirdly, due to data limitations, we are unable to explore the potential mechanisms behind the results of this study. However, we believe that a reduction in healthcare burdens and an increase in risk-taking capacity could be potential mechanisms. On the one hand, after the implementation of IMIS, migrant workers can receive higher reimbursements when they fall ill due to the improved reimbursement rates. On the other hand, the reduced economic risks associated with illness may make migrant workers more willing to take entrepreneurial risks. Nevertheless, these mechanisms would require further investigation in future study.

## 7 Conclusion and policy implications

In this study, we use panel data from CHFS from 2013 to 2019 to empirically test the effect of the IMIS on migrant workers’ return-to-hometown entrepreneurship by employing a time-varying DID method. The main conclusions are as follows: Firstly, we find that the IMIS significantly increases the likelihood of migrant workers returning to their hometowns for entrepreneurship by 0.44%, which remains valid after a series of robustness checks, including DIDM, endogeneity, alternative models, and placebo tests. Secondly, this effect is more pronounced for those who are male and with lower educational levels, higher income, larger social networks, and lower risk preferences. Thirdly, the policy synergy of MEI and IMIS can yield greater effects on the return of migrant workers to their hometowns for entrepreneurship.

This paper provides the following policy implications: Firstly, policymakers should concentrate on further enhancing the healthcare support system in rural areas. This involves a continuous extension of medical insurance coverage and a judicious augmentation of welfare benefits for migrant workers, intending to alleviate the financial strain associated with potential medical expenditures during the entrepreneurial journey and foster heightened entrepreneurial motivation. Secondly, the government and society should pay attention to the medical protection of the key groups of rural migrant workers, and formulate different details of the medical insurance system for different situations, so that the entrepreneurial environment of the rural migrant workers can be more effectively protected. In addition, it is necessary to guide and organize migrant workers to actively participate in urban and rural residents’ basic medical insurance, so as to make up for the short board of medical insurance for migrant workers and effectively improve their medical insurance treatment. Thirdly, it is recommended that relevant authorities bolster risk education initiatives for rural migrant workers, facilitating their rational understanding of risks and augmenting their capacity to manage and mitigate them. Furthermore, it is crucial to cultivate collaboration between governments and social organizations at various levels, aiming to construct social network “bridges” for rural migrant workers aspiring to return to their hometowns and embark on entrepreneurial ventures. Additionally, it is essential to encourage migrant workers to actively participate in various social activities, which will help to broaden their social network resources and ultimately promote their entrepreneurship in their respective hometowns. Lastly, the optimization of a system should take into account the systemic and holistic nature (84). Policymakers, in their drive to promote rural revitalization, should consider medical security and entrepreneurship support as an integrated whole to achieve the maximum policy effectiveness.

Our research findings also carry implications for other developing countries with similar welfare program improvements and efforts aimed at rebalancing urban and rural population migration structures. Over the past decade, China has continuously enhanced the welfare provisions within its rural labor market plan. Consequently, we anticipate that the impact of this program on labor supply has become increasingly prominent in recent years. However, it is essential to note that the expected effects of certain welfare programs have yet to be rigorously analyzed. As such, it is crucial that future research seeks to validate the individual and combined effects of these new policies using updated and more robust datasets. This will enable a more comprehensive understanding of the influence of welfare improvements on labor dynamics and the broader socio-economic landscape in developing countries.

## Data availability statement

Publicly available datasets were analyzed in this study. This data can be found at: China Household Finance Survey (CHFS), https://chfs.swufe.edu.cn/index.htm.

## Author contributions

PH: Conceptualization, Funding acquisition, Resources, Writing – original draft. ZS: Data curation, Formal analysis, Methodology, Software, Supervision, Validation, Visualization, Writing – original draft, Writing – review & editing. LL: Writing – original draft. JL: Supervision, Validation, Visualization, Writing – review & editing.

## Footnotes

^1^ https://www.gov.cn/gongbao/content/2016/content_5036268.htm

^2^ http://bj.bendibao.com/zffw/20171214/246632.shtm

^3^ http://www.gov.cn/xinwen/2017-11/12/content_5239129.htm

^4^ http://www.chinanews.com/gn/2016/10-10/8025768.shtml

^5^ http://www.gov.cn/xinwen/2017-12/19/content_5248363.htm

^6^ https://www.gov.cn/xinwen/2019-12/16/content_5461646.htm

^7^ https://www.gov.cn/xinwen/2020-07/17/content_5527709.htm

^8^For instance, “Opinions on Promoting High-Quality Development of Returning to Hometown and Entrepreneurship,” jointly issued by the National Development and Reform Commission and 19 other departments in 2020, and “Opinions on Ensuring the Current Employment and Entrepreneurship of Migrant Workers,” jointly issued by the Ministry of Human Resources and Social Security and the National Development and Reform Commission, along with 15 other departments.

^9^The rationale behind this approach is grounded in the general observation that an individual’s risk preference reflects their personality traits, which typically do not undergo drastic changes. Hence, our hypothesis is predicated on the assumption that an individual’s risk preference remains stable, barring any significant shocks. To validate the consistency of risk preference, we also compared the same individual’s risk preferences in 2015 and 2019. The results indicate that only 824 samples exhibited changes in risk preference between 2015 and 2019, constituting approximately 3.8% of the 2015 sample size. Upon examination, it was found that excluding these samples does not materially affect the principal findings of this study.

^10^The choice of “self-employed, operating an individual or private business, or running an online store” as proxies for entrepreneurship is justified in the survey by these options embodying the essential characteristics of entrepreneurship, including autonomy, innovation, and risk-taking. These roles typically involve full personal control and management of a business, entail certain operational risks, and are often pursued to achieve personal career goals and financial independence. Additionally, these activities reflect the entrepreneurial spirit, such as independent thinking, innovative capability, and self-motivation.

^11^The rationale for categorizing education level as an ordinal variable stems from two main considerations. Firstly, the dataset encompasses a spectrum of nine distinct options, spanning from illiteracy to doctoral degrees. Secondly, this classification into nine categories allows for a more nuanced reflection of individual differences in educational levels, thereby enabling a more effective control for potential impacts arising from diverse stages of educational experiences on the outcomes examined in this study.

^12^These pensions come from government or public institution retirement pensions.

^13^These social insurance programs include Urban Employee Basic Medical Insurance (UEBMI), URBMI, NRCMS, URRBMI, and Free Medical Care (FMC).

^14^*Renqing* is a concept of significant importance in Chinese culture regarding social interactions and interpersonal relationships. It typically refers to the acts of mutual assistance, care, respect, and politeness displayed by individuals in the context of social exchanges. These behaviors play a crucial role in establishing and maintaining positive interpersonal relationships. *Renqing* encompasses activities such as the exchange of gifts, hosting meals, the giving of symbolic presents, and offering assistance, all of which serve as expressions of gratitude, respect, and friendship.

^15^The Rural Revitalization Strategy is a comprehensive initiative launched by the Chinese government in 2018, aimed at fostering economic development in rural areas, improving the living standards of rural residents, and achieving balanced development between urban and rural regions.

^16^The Probit model is based on the cumulative normal distribution function. It assumes that the underlying process generating the binary outcome follows a normal distribution. In the Probit model, the probability of the outcome variable equaling a particular value (usually 1) is modeled as a function of a normal cumulative distribution of the independent variables.

^17^The Logit model, on the other hand, is based on the logistic function. It uses the logistic distribution to model the probability of the binary outcome. The logistic function in the Logit model transforms the linear combination of the independent variables, providing a probability that ranges between 0 and 1.

^18^We also divide the total sample into two sub-samples based on the median age (40 years) of our sample. The results reveal that the effects of IMIS are 0.0047 (*p* < 0.05) for individuals aged under 40 and 0.0043 (*p* < 0.1) for those aged over 40. Both of these effects are statistically significant. However, this does not affect our interpretation of the age heterogeneity results.

^19^In China, compulsory education spans 9 years, culminating with the completion of junior high school. This signifies that individuals, upon concluding compulsory education, have the option to voluntarily pursue further studies or enter the workforce. Therefore, our categorization of high and low education is based on the completion of compulsory education. Nonetheless, higher education also typically denotes the attainment of a bachelor’s degree or higher, and thus, we conduct additional analysis using this criterion. The outcomes align closely with the results depicted in Table 5, panel c.

^20^According to the contents of the questionnaire, expenditures related to social interactions include discretionary spending during holidays (e.g., the Spring Festival and Mid-Autumn Festival), due to celebratory events (e.g., weddings and funerals), for education, for healthcare, for living, and for other purposes.

^21^ https://www.stats.gov.cn/xxgk/sjfb/zxfb2020/202302/t20230228_1919001.html?eqid=fa9f347f0008349f00000002645671cd
